# Supplementary material for: COZOID: contact zone identifier for visual analysis of protein-protein interactions
Source: BMC Bioinformatics. 2018 Apr 6;19:125. doi: 10.1186/s12859-018-2113-6 (PMC5889581; doi:10.1186/s12859-018-2113-6)
Supplement: Supplementary file 2 — Software build. Executable binary file of the software tool. (ZIP 118,784 kb) [file 12859_2018_2113_MOESM2_ESM.zip › cozoid/platform/docs/testng-6.5.1-javadoc.zip!/overview-tree.html]

Class Hierarchy (TestNG)


---


|  |  |  |
| --- | --- | --- |
| **Overview** \| Package \| Class \| **Tree** \| **Deprecated** \| **Index** \| **Help** | |  |
| PREV   NEXT | **FRAMES**    **NO FRAMES**     **All Classes** |  |


---


## Hierarchy For All Packages

**Package Hierarchies:**: org.testng, org.testng.annotations, org.testng.xml

---

## Class Hierarchy

- java.lang.Object
  - org.testng.internal.junit.ArrayAsserts
    - org.testng.**AssertJUnit**
    - org.testng.**Assert**- org.testng.**ClassMethodMap**- org.testng.**CommandLineArgs**- org.testng.**ConversionUtils**- org.testng.**Converter**- org.testng.**DependencyMap**- org.testng.**FileAssert**- org.apache.tools.ant.ProjectComponent (implements java.lang.Cloneable)
                    - org.apache.tools.ant.Task
                      - org.testng.**TestNGAntTask**
                    - org.apache.tools.ant.taskdefs.PumpStreamHandler (implements org.apache.tools.ant.taskdefs.ExecuteStreamHandler)
                      - org.testng.**TestNGAntTask.TestNGLogSH**
                      - org.testng.**Reporter**- org.testng.**ReporterConfig**- org.testng.**ReporterConfig.Property**- org.testng.**SuiteRunner** (implements org.testng.IInvokedMethodListener, org.testng.ISuite, java.io.Serializable)- org.testng.**SuiteRunnerWorker** (implements org.testng.internal.thread.graph.IWorker<T>)- org.testng.**SuiteRunState** (implements java.io.Serializable)- org.testng.**TestListenerAdapter** (implements org.testng.internal.IResultListener2)- org.testng.**TestNG**- org.testng.**TestNG.ExitCodeListener** (implements org.testng.internal.IResultListener2)- org.testng.**TestNGUtils**- org.testng.**TestRunner** (implements org.testng.ITestContext, org.testng.internal.ITestResultNotifier, org.testng.internal.thread.graph.IThreadWorkerFactory<T>)- java.lang.Throwable (implements java.io.Serializable)
                                              - java.lang.Exception
                                                - java.lang.RuntimeException
                                                  - org.testng.**SkipException**
                                                    - org.testng.**TimeBombSkipException**
                                                    - org.testng.**TestNGException**
                                                      - org.testng.**TestException**
                                              - org.testng.xml.**XmlClass** (implements java.lang.Cloneable, java.io.Serializable)- org.testng.xml.**XmlInclude** (implements java.io.Serializable)- org.testng.xml.**XmlMethodSelector**- org.testng.xml.**XmlPackage** (implements java.io.Serializable)- org.testng.xml.**XmlSuite** (implements java.lang.Cloneable, java.io.Serializable)- org.testng.xml.**XmlTest** (implements java.lang.Cloneable, java.io.Serializable)- org.testng.xml.**XmlUtils**

## Interface Hierarchy

- java.lang.Cloneable
  - org.testng.**ITestNGMethod** (also extends java.lang.Comparable<T>, java.io.Serializable)
  - java.lang.Comparable<T>
    - org.testng.**ITestNGMethod** (also extends java.lang.Cloneable, java.io.Serializable)- org.testng.**ITestResult** (also extends org.testng.IAttributes)
    - org.testng.annotations.**IAnnotation**
      - org.testng.annotations.**IConfigurationAnnotation**- org.testng.annotations.**IDataProviderAnnotation**- org.testng.annotations.**IExpectedExceptionsAnnotation**- org.testng.annotations.**IFactoryAnnotation** (also extends org.testng.internal.annotations.IDataProvidable, org.testng.annotations.IParameterizable)- org.testng.annotations.**IObjectFactoryAnnotation**- org.testng.annotations.**IParameterizable**
                  - org.testng.annotations.**IConfigurationAnnotation**- org.testng.annotations.**IFactoryAnnotation** (also extends org.testng.internal.annotations.IDataProvidable)- org.testng.annotations.**ITestAnnotation** (also extends org.testng.internal.annotations.IDataProvidable, org.testng.annotations.ITestOrConfiguration)- org.testng.annotations.**ITestOrConfiguration**
                          - org.testng.annotations.**IConfigurationAnnotation**- org.testng.annotations.**ITestAnnotation** (also extends org.testng.internal.annotations.IDataProvidable)
                  - org.testng.annotations.**IParametersAnnotation**- org.testng.annotations.**ITestAnnotation** (also extends org.testng.internal.annotations.IDataProvidable, org.testng.annotations.ITestOrConfiguration)- org.testng.annotations.**ITestOrConfiguration**
                        - org.testng.annotations.**IConfigurationAnnotation**- org.testng.annotations.**ITestAnnotation** (also extends org.testng.internal.annotations.IDataProvidable)
      - org.testng.**IConfigureCallBack**- org.testng.internal.annotations.IDataProvidable
          - org.testng.annotations.**IFactoryAnnotation** (also extends org.testng.annotations.IParameterizable)- org.testng.annotations.**ITestAnnotation** (also extends org.testng.annotations.ITestOrConfiguration)
          - org.testng.**IHookCallBack**- org.testng.**IInstanceInfo**- org.testng.**IInvokedMethod**- org.testng.**IMethodInstance**- org.testng.**IMethodSelectorContext**- org.testng.**IModuleFactory**- org.testng.**IRetryAnalyzer**- org.testng.**ITest**- org.testng.**ITestClassFinder**- org.testng.**ITestMethodFinder**- org.testng.**ITestNGListener**
                                - org.testng.**IAnnotationTransformer**
                                  - org.testng.**IAnnotationTransformer2**
                                  - org.testng.**IAnnotationTransformer2**- org.testng.**IConfigurable**- org.testng.**IConfigurationListener**
                                        - org.testng.**IConfigurationListener2**
                                        - org.testng.**IConfigurationListener2**- org.testng.**IExecutionListener**- org.testng.**IHookable**- org.testng.**IInvokedMethodListener**
                                                - org.testng.**IInvokedMethodListener2**
                                                - org.testng.**IInvokedMethodListener2**- org.testng.**IMethodInterceptor**- org.testng.**IReporter**- org.testng.**ISuiteListener**- org.testng.**ITestListener**
                                - org.testng.**ITestNGListenerFactory**- org.testng.**ITestRunnerFactory**- java.io.Serializable
                                      - org.testng.**IAttributes**
                                        - org.testng.**ISuite**- org.testng.**ITestContext**- org.testng.**ITestResult** (also extends java.lang.Comparable<T>)
                                        - org.testng.**IClass**
                                          - org.testng.**ITestClass** (also extends java.io.Serializable)
                                          - org.testng.**IExtraOutput**- org.testng.**IMethodSelector**- org.testng.**IResultMap**- org.testng.**ISuiteResult**- org.testng.**ITestClass** (also extends org.testng.IClass)- org.testng.**ITestNGMethod** (also extends java.lang.Cloneable, java.lang.Comparable<T>)- org.testng.**ITestObjectFactory**
                                                        - org.testng.**IObjectFactory**- org.testng.**IObjectFactory2**

## Annotation Type Hierarchy

- org.testng.annotations.**AfterClass** (implements java.lang.annotation.Annotation)- org.testng.annotations.**AfterGroups** (implements java.lang.annotation.Annotation)- org.testng.annotations.**AfterMethod** (implements java.lang.annotation.Annotation)- org.testng.annotations.**AfterSuite** (implements java.lang.annotation.Annotation)- org.testng.annotations.**AfterTest** (implements java.lang.annotation.Annotation)- org.testng.annotations.**BeforeClass** (implements java.lang.annotation.Annotation)- org.testng.annotations.**BeforeGroups** (implements java.lang.annotation.Annotation)- org.testng.annotations.**BeforeMethod** (implements java.lang.annotation.Annotation)- org.testng.annotations.**BeforeSuite** (implements java.lang.annotation.Annotation)- org.testng.annotations.**BeforeTest** (implements java.lang.annotation.Annotation)- org.testng.annotations.**Configuration** (implements java.lang.annotation.Annotation)- org.testng.annotations.**DataProvider** (implements java.lang.annotation.Annotation)- org.testng.annotations.**ExpectedExceptions** (implements java.lang.annotation.Annotation)- org.testng.annotations.**Factory** (implements java.lang.annotation.Annotation)- org.testng.annotations.**Guice** (implements java.lang.annotation.Annotation)- org.testng.annotations.**Listeners** (implements java.lang.annotation.Annotation)- org.testng.annotations.**NoInjection** (implements java.lang.annotation.Annotation)- org.testng.annotations.**ObjectFactory** (implements java.lang.annotation.Annotation)- org.testng.annotations.**Optional** (implements java.lang.annotation.Annotation)- org.testng.annotations.**Parameters** (implements java.lang.annotation.Annotation)- org.testng.annotations.**Test** (implements java.lang.annotation.Annotation)- org.testng.annotations.**TestInstance** (implements java.lang.annotation.Annotation)

## Enum Hierarchy

- java.lang.Object
  - java.lang.Enum<E> (implements java.lang.Comparable<T>, java.io.Serializable)
    - org.testng.**TestNGAntTask.Mode**

---


|  |  |  |
| --- | --- | --- |
| **Overview** \| Package \| Class \| **Tree** \| **Deprecated** \| **Index** \| **Help** | |  |
| PREV   NEXT | **FRAMES**    **NO FRAMES**     **All Classes** |  |


---
